# Book Reviews

**Published:** 1893-01

**Authors:** 


### Book Reviews.

A MANUAL OF THE PRACTICE OF MEDICINE, prepared especially for students.—By A. A. Stevens, A. M., M D., Instructor of Physical Diagnosis in the University of Pennsylvania and Demonstrator of Pathology in the Woman's Medical College, Philadelphia. Illustrated. Price $2.50 Philadelphia: W. B. Saunders, 913 Walnut street, 1893.

WHILE it is not best that students of medicine should depend too much upon manuals and quiz compends to the exclusion of their regular text-books, at the same time we believe they have their sphere and are of great benefit to the student preparing for his final examination, for hospital appointment, and as a reference book. Pope says, "Half our knowledge we must snatch, not take." This is true, in a great measure, of medical knowledge as taught in most of the medical schools of to-day. This little manual deals very thoroughly in a concise manner with all the principal diseases the physician is called upon to treat, and is, in fact, one of the most complete of its kind. C. E. J.

---

The "American Text-Book of Surgery," edited by Professors Keen and White of Philadelphia, which has only been issued a few months, is already a phenomenal success. It has been adopted as a text-book by forty-nine of our leading medical colleges and universities. Nearly five thousand copies have been placed in physician's libraries, and every indication points to a sale of at least as many copies more in the next six months.

Dr. Nicholas Senn, of Chicago, is now preparing a "Syllabus of Lectures on the Practice of Surgery," arranged in conformity with the "American Text-Book of Surgery," which will be a valuable aid to all who have this great book.

---

The Messrs. Macmillan & Co. announce that the recently completed edition of Foster's Text-book of Physiology in four parts is to be supplemented by the issue of an appendix on "The Chemical Basis of the Animal Body," by A. Sheridan Lea, Sc. D., F. R. S. Dr. Lea is Lecturer on Physiology to the University of Cambridge, England.

